# Validation of the Maslach Burnout Inventory-General Survey 9-item short version: psychometric properties and measurement invariance across age, gender, and continent

**DOI:** 10.3389/fpsyg.2024.1439470

**Published:** 2024-07-16

**Authors:** Anni Wang, Yinfei Duan, Peter G. Norton, Michael P. Leiter, Carole A. Estabrooks

**Affiliations:** ^1^School of Nursing, Fudan University, Shanghai, China; ^2^Faculty of Nursing, University of Alberta, Edmonton, AB, Canada; ^3^Department of Family Medicine, Cumming School of Medicine, University of Calgary, Calgary, AB, Canada; ^4^Department of Psychology, Acadia University, Wolfville, NS, Canada

**Keywords:** burnout, MBI-GS, validity, reliability, Rasch analysis

## Abstract

**Background:**

The Maslach Burnout Inventory-General Survey (MBI-GS) stands as the preeminent tool for assessing burnout across various professions. Although the MBI-GS9 emerged as a derivative of the MBI-GS and has seen extensive use over several years, a comprehensive examination of its psychometric properties has yet to be undertaken.

**Methods:**

This study followed the Standards for Educational and Psychological Testing guidelines to validate the MBI-GS9. Employing a combined approach of classical test theory and item response theory, particularly Rasch analysis, within an integrated framework, the study analyzed data from 16,132 participants gathered between 2005 and 2015 by the Centre for Organizational Research at Acadia University.

**Results:**

The findings revealed that the MBI-GS9 exhibited satisfactory reliability and validity akin to its predecessor, the MBI-GS. Across its three dimensions, Cronbach’s α and omega coefficients ranged from 0.84 to 0.91. Notably, the MBI-GS9 displayed no floor/ceiling effects and demonstrated good item fit, ordered threshold, acceptable person and item separation and reliability, clear item difficulty hierarchy, and a well-distributed item threshold. However, the results suggested a recommended minimum sample size of 350 to mitigate potential information loss when employing the MBI-GS9. Beyond this threshold, the observed mean difference between the MBI-GS and MBI-GS9 held minimal practical significance. Furthermore, measurement equivalence tests indicated that the MBI-GS9 maintained an equivalent three-factor structure and factor loadings across various gender, age, and continent groups, albeit with inequivalent latent values across continents.

**Conclusion:**

In sum, the MBI-GS9 emerges as a reliable and valid alternative to the MBI-GS, particularly when utilized within large, diverse samples across different age and gender demographics. However, to address potential information loss, a substantial sample size is recommended when employing the MBI-GS9. In addition, for cross-cultural comparisons, it is imperative to initially assess equivalence across different language versions at both the item and scale levels.

## 1 Introduction

Burnout is a psychological syndrome resulting from prolonged exposure to chronic workplace stress, characterized by three main dimensions: overwhelming exhaustion, feelings of cynicism and detachment from the job, and a sense of ineffectiveness and lack of accomplishment (Leiter and Schaufeli, 1996; Maslach and Leiter, 2016). It is linked to various negative work-related outcomes, including job dissatisfaction, low organizational commitment, and turnover (Kumar et al., 2007; Leiter and Maslach, 2009; Maslach and Leiter, 2016; McCormack et al., 2018). In addition, burnout is associated with serious health issues such as cardiovascular disease and type 2 diabetes (Melamed et al., 2006; Toppinen-Tanner et al., 2009; Shirom et al., 2013). Due to its high prevalence and adverse effects, the World Health Organization (WHO) has declared burnout an occupational public health concern and urged actions to combat it (World Health Organization, 2019). The significant personal and organizational impacts of burnout underscore the need for early detection and intervention, highlighting the importance of a validated and efficient measurement tool (Maslach and Leiter, 2016).

The Maslach Burnout Inventory (MBI) is one of the most widely used measures of burnout, validated through extensive research over the past 35 years (Maslach and Leiter, 2016; McCormack et al., 2018; Shoman et al., 2021). The original 22-item MBI was developed for various human service occupations, and alternative versions have been created for different fields. Notably, the MBI-General Survey (MBI-GS) was designed with more “occupation-neutral” items. In this version, the dimension of depersonalization was expanded to reflect a broader sense of negative detachment from work and renamed “cynicism,” while the dimension of personal accomplishment was broadened and renamed “professional efficacy” (Schaufeli, 1996; Maslach and Leiter, 2016). This made the MBI-GS a more universally applicable tool for both research and practice across different occupations. The MBI-GS has been widely adopted and translated into multiple languages, including French, Spanish, Polish, and Dutch, among others (Bravo et al., 2021).

The MBI-GS, with its 16 items, takes 5–10 min to complete, making it shorter than the original 22-item MBI. Nevertheless, some studies seek even more abbreviated versions to lessen respondent burden while maintaining reliability and validity. Examples include one-item (Nagasaki et al., 2022) and two-item (Rohland et al., 2004; West et al., 2012; Chohan et al., 2021) versions, which have shown satisfactory reliability and concurrent validity against the MBI-GS. However, these shorter versions do not include the dimension of professional efficacy and have only been validated among health professionals (Rohland et al., 2004; West et al., 2012; Chohan et al., 2021; Nagasaki et al., 2022). Given the multidimensional nature of burnout, a short version of the MBI-GS that includes all three dimensions is necessary to preserve theoretical comprehensiveness. From a statistical perspective, it is recommended to have a minimum of two or three observed indicators for a latent variable (Bollen, 2002; Silva et al., 2006). If fewer than three, the measure may fail to capture the actual phenomenon or latent variable thoroughly and tend to show poor fit in confirmatory factor analysis (Silva et al., 2006). Thus, a 9-item version could be a promising short option for the MBI-GS, retaining most of the test information with fewer items. By selecting three items from each dimension, the developers of the original MBI-GS created the 9-item short version (Leiter, 2006). Although the MBI-GS9 has been used in various studies, only one study has compared it to the Spanish 16-item MBI-GS in Spanish teachers (Merino-Soto et al., 2023). According to the developers, the nine items were selected based on the lowest residual covariance between items (Leiter, 2006). However, this residual covariance may be idiosyncratic to the sample and may not generalize to other populations (Merino-Soto et al., 2023). A short version of the MBI-GS would be valuable in practice, especially in complex surveys (e.g., longitudinal or cross-sectional studies with multiple measures) (McManus et al., 2002; Melamed et al., 2006; Toppinen-Tanner et al., 2009; Chamberlain et al., 2017), where a shorter scale is crucial for maintaining a high response rate. While studies using the MBI-9 indicate good reliability for its three subscales, a thorough evaluation of its psychometric properties is essential to endorse its application in practice and research.

Research question: Does the MBI-GS9 maintain good and comparable psychometric properties against the original 16-item MBI-GS?

### 1.1 Measurement invariance of the MBI-GS9 across continent, age, and gender

Measurement invariance provides evidence of equivalence for generalizing an instrument (American Educational Research Association, 2014). In evaluating measurement invariance, it is essential to first test the factor structure, which means the instrument must have the same latent structure across different samples. Following the factor structure, it is recommended to test measurement weights (metric invariance) and measurement intercepts (scalar invariance) to determine whether factor loadings and latent means are equivalent across different groups (Vandenberg and Lance, 2000). To generalize the MBI-GS9 across the world, a large, international sample with a variety of occupations is needed to compare different age, gender, and global cultural context (e.g., aggregated into continents in this study) groups.

While the measurement invariance of the MBI-GS9 remains to be tested, the original MBI-GS has mostly been tested for measurement invariance across cultures, countries, and language-speaking areas (Marais et al., 2009; Fernández-Arata et al., 2015; Juarez Garcia et al., 2020; Bravo et al., 2021), as well as age, gender, and occupation in a few studies (Fernández-Arata et al., 2015; Juarez Garcia et al., 2020; Bravo et al., 2021). The measurement equivalence across age has been supported in Colombian workers (Bravo et al., 2021), Latin-American teachers (Juarez Garcia et al., 2020), Peruvian workers (Fernández-Arata et al., 2015), and Colombian workers (Bravo et al., 2021) across gender. As to measurement invariance across cultures, the MBI-GS has shown a stable three-factor structure in Latin America (Fernández-Arata et al., 2015), South Africa (Marais et al., 2009), Colombia (Bravo et al., 2021), and Latin-American countries separately (Juarez Garcia et al., 2020). A three-nation comparison of the MBI-GS among Finnish, Swedish, and Dutch samples showed non-invariance at the item level—recording low factor loadings and item error correlations (Schutte et al., 2000). Dutch and Greek samples showed invariance in a few items (Xanthoupoulou et al., 2013), so did in the comparison between U.S. and Jamaican samples (Denton et al., 2013). The above studies helped to draw the hypotheses below:

*H1*: The MBI-GS9 has equivalent structure, measurement weights, and measurement intercepts across age and gender.

*H2*: The MBI-GS9 has equivalent structure and measurement weights (2a) but may not have equivalent measurement intercepts (2b) across continents.

### 1.2 Combining CTT and Rasch analysis under the standards

Recent progress in strategies for validating shortened scales recommends combining classical test theory (CTT) and item response theory (IRT) (Sébille et al., 2010). Although CTT has been used for many decades, it has drawbacks because many statistics are omnibus and sample-dependent (Sébille et al., 2010). Rasch analysis, or the one-parameter logistic model—a type of IRT model (Kline, 2005)—has been widely used in life sciences recently, offering several advantages (Stolt et al., 2022).

First, compared to CTT, Rasch analysis is sample-independent and focuses on how each item relates to the construct (Jabrayilov et al., 2016). This provides more informative item-level psychometric properties than tests of the entire parameter or subscale (Meade and Kroustalis, 2006). Second, unlike traditional IRT, Rasch analysis provides a variety of item properties (e.g., item difficulty and item separation index) and a clear set of criteria to mitigate subjective judgment (Stolt et al., 2022). Third, and most importantly, Rasch analysis offers more precise and accurate measurements of both persons and items, supporting various aspects of reliability or precision (Kline, 2005). While the validation of the MBI-GS9 in healthcare practitioners (Riley et al., 2018) and Spanish teachers (Merino-Soto et al., 2023) has shown satisfactory psychometric properties based on CTT, item functioning has not been evaluated. A few studies have indicated item-level differences in some MBI-GS items (Denton et al., 2013; Juarez Garcia et al., 2020), some of which are included in the MBI-GS9.

Studies solely using Rasch analysis have limitations in providing a robust, comprehensive, and precise reporting of methodological choices and results (Juarez Garcia et al., 2020). An integrated framework that combines CTT and Rasch analysis could address these limitations. The Standards for Educational and Psychological Testing (the Standards) are regarded as best practice in the field of psychometrics (Streiner et al., 2015). These standards provide criteria for the development and evaluation of tests, emphasizing that validation involves accumulating relevant evidence rather than distinct types of reliability and validity (Downing, 2003). Hence, this study presents a pioneering application of combining CTT and Rasch analysis under the framework of the Standards (American Educational Research Association, 2014). Our findings will provide valuable insights for burnout researchers globally and advance the field’s understanding of scale development.

### 1.3 The present study

By leveraging the benefits of both CTT and Rasch analysis under the framework of the Standards, this study aims to validate the MBI-GS9 using a large international sample. The study seeks to answer and test the previously identified research question and hypotheses. This research will extend our knowledge of burnout measurement in three important ways:

1. Comprehensive Evidence of Validity and Reliability: By collecting comprehensive evidence of validity and reliability, the results will inform researchers and practitioners about the suitability of using either the MBI-GS9 or the MBI-GS. This will help in making informed decisions when choosing the appropriate tool for different contexts.
2. Testing Measurement Invariance: By testing the measurement invariance of the MBI-GS9 across gender, age, and continent, we will illustrate the extent to which this instrument can be used across a variety of groups. This will lay the foundation for future comparative studies and ensure that the MBI-GS9 is applicable in diverse settings.
3. Demonstrating an Integrated Validation Approach: By combining CTT and Rasch analysis under the framework of the Standards, this study will demonstrate an integrated approach for validating a measurement tool. In addition, it will provide specific item-level properties that will be useful for shortening other MBI family members in future. This approach can serve as a model for validating other psychometric instruments and enhancing the rigor and applicability of measurement tools in various fields.

Overall, this study aims to enhance the understanding and application of the MBI-GS9, providing valuable insights for burnout researchers and practitioners worldwide.

## 2 Methods

### 2.1 Participants

We used data collected by the Centre for Organizational Research & Development (COR&D) at Acadia University between 2005 and 2015. This center, the birthplace of the MBI, was established in 1991 to examine organizational relationships through topics such as burnout, work engagement, work/life balance, civility in the workplace, and managing change. Researchers who requested permission to officially translate and use the MBI-GS in research projects were asked to provide de-identified data in return. Since we utilized secondary data, we were unable to eliminate the risk of careless responses at the survey stage. However, we took several steps to mitigate this risk before conducting data analysis. First, we found the amount of missing data for all items of the MBI-GS was approximately 2%, and the missing data appeared to be completely random. The Rasch model typically handles such missing data by automatically excluding it. Second, the D^2^ distance method was employed to identify inconsistent response patterns and identify multivariate outliers (Curran, 2016). The sample used in the current study comprised 16,132 participants out of 24,613 cases.

A total of 11,632 participants were used in the analysis, with the characteristics of the participants shown in Table 1. There were 10,318 (62.0%) female participants, with the majority aged 40–49 (29.6%). Participants were from North America (22.6%), Europe (34.9%), Asia (29.0%), and other continents (13.5%). This diversity in gender, age, and continent allowed us to test the measurement invariance of the MBI-GS9 across these characteristics.

**Table 1:** Socio-demographic characteristics of the participants (*n* = 11,632).

Variables		N (%)
Gender	Male	6,314(38.0)
	Female	10,318(62.0)
Age	18–29	2,672(16.6)
	30–39	4,865(29.3)
	40–49	4,927(29.6)
	50–59	3,176(19.1)
	60+	902(5.4)
Continent	North America	3,752(22.6)
	Europe	5,797(34.9)
	Other	2,253(13.5)
	Asia	4,830(29.0)
Time in the job	<1 year	612(3.7)
	1–5 years	3,192(19.2)
	6–10 years	491(3.0)
	11–20 years	698(4.2)
	>20 years	543(3.3)
	missing	11,096(66.7)
Working site	Public service/retail	101(0.6)
	Post office	336(2.2)
	hospital	3,910(23.5)
	University	1,511(9.1)
	University library	313(1.9)
	Missing	10,431(62.7)
Profession	Salvation army	231(1.4)
	Blue Collar	296(1.8)
	Hospital staff	375(2.3)
	White collar (profit)	1,639(9.9)
	Civil servant	12 (0.1)
	Nurse	1,401(8.4)
	Doctor	211(1.3)
	University staff	759(4.6)
	Engineer/technician	346(2.1)
	Police officer	2,443(14.7)
	Teacher	314(1.9)
	Manager	365(2.2)
	Social worker psychologist	43(0.3)
	White collar (non-profit)	416(2.5)
	Dentists	829(5.0)
	Patients burnout	57(0.3)
	Government	105(0.6)
	Judge	391(2.4)
	Other (Famer, Entrepreneurs, etc.)	14(0.1)
	Missing	6,385(38.4)

### 2.2 Measures

We used a number of socio-demographic variables, including age, gender, continent, time in the job, working site, and profession. Although participating groups were requested to use a standard format to report demographic information on participants and descriptions of the surveyed organizations, much of the accompanying information was missing in many instances.

#### 2.2.1 The MBI-GS

Developed from the original MBI designed for human service occupations, the Maslach Burnout Inventory-General Survey (MBI-GS) consists of 16 items rated on a 7-point scale ranging from 0 (never) to 6 (daily) (Maslach et al., 1996). The scale is composed of three subscales: exhaustion (5 items), cynicism (5 items), and professional efficacy (6 items). Burnout is reflected in higher scores on exhaustion and cynicism and lower scores on professional efficacy. The score for each subscale is obtained by dividing the total score of all items in that subscale by the number of items. The MBI-GS has demonstrated high validity across different cultural contexts, with the reliability of each subscale ranging from 0.85 to 0.89 (Maslach and Jackson, 1981). For the projects included in this study, translations of the MBI-GS into the local language were used when the language was other than English.

#### 2.2.2 The MBI-GS9

This abbreviated version was derived by selecting three items from each dimension of the MBI-GS, resulting in a 9-item burnout measure. It includes the emotional exhaustion subscale (Ex: items 3, 4, and 5), cynicism (Cyn: 2, 3, and 5), and professional efficacy (Ef: 3, 4, and 5) from the MBI-GS. The response scale maintains the same seven options, ranging from 0 (Never) to 6 (Daily). The MBI-GS9 has demonstrated good reliability and validity in Spanish teachers (Merino-Soto et al., 2023), as well as in other empirical studies (McManus et al., 2002; Chamberlain et al., 2017).

#### 2.2.3 The area of worklife survey

The AWS is a 29-item instrument designed to assess six areas of work life: manageable workload, controllability, rewards, sense of community, fairness, and congruence of values (Leiter and Maslach, 2003; Leiter et al., 2010). These items were developed from a series of staff surveys conducted by the Centre for Organizational Research and Development as a means of evaluating these aspects of work life (Leiter and Harvie, 1998). Each subscale comprises both positively worded items reflecting congruence (e.g., manageable workload: “I have enough time to do what’s important in my job”) and negatively worded items reflecting incongruence (e.g., values: “Working here forces me to compromise my values”). Respondents rate their agreement with these statements on a 5-point scale ranging from 1 (strongly disagree) to 5 (strongly agree). Negatively worded items are reverse-scored, with higher scores indicating greater congruence, namely a higher degree of perceived alignment between the workplace and the respondent’s preferences. The scale has consistently demonstrated a stable factor structure and reliability across various samples (Leiter and Maslach, 2003). According to the model of six areas of work life (AWL), burnout is viewed as a prolonged response to chronic interpersonal stressors in six domains: workload, control, reward, community, fairness, and values (Leiter and Maslach, 1999). The AWL framework suggests that misalignment in these areas may contribute to work attrition (Leiter and Harvie, 1998). Higher congruence indicates a greater perceived alignment between the workplace and the individual (Leiter and Maslach, 2003). In analyses involving the AWS, the analytic sample consisted of 3,802 participants who completed the survey. The reliability of each subscale in this study ranged from 0.7 to 0.8.

### 2.3 Data analysis

This study was conducted and reported in accordance with the guidelines and recommendations for reporting the results of studies of instrument and scale development and testing (Streiner and Kottner, 2014). Data analysis was performed using SPSS 21.0, Amos 29.0.0 (Chicago, IL, United States), and WINSTEPS 5.6.3.^1^ Statistical description was carried out using means, standard deviations (SDs), frequency, and percentage. For Rasch analysis, we employed the unrestricted Partial Credit Rasch model as it is suitable for analyzing polytomous items with ordered categories (Masters, 1982). Following the Standards, the assessment of the psychometric properties of a new instrument involves collecting evidence on (1) reliability/precision; (2) validity; and (3) fairness/equivalence (American Educational Research Association, 2014). We framed all evidence from classical test theory (CTT) and Rasch analysis under the Standards, which is described below and summarized in Table 1. This comprehensive approach ensures a thorough evaluation of the instrument’s properties and enhances the credibility and robustness of the findings (Table 2).

**Table 2:** Psychometric properties evidence framework.

Aspect of evidence	Analysis	Evidence	Criteria/Threshold
Reliability/precision	CTT	Cronbach’s α coefficient and omega coefficient	>0.7
		Floor/ceiling effect	>20% of respondents report either the best or lowest score.
	Rasch analysis	Item fit (infit MNSQ, outfit MNSQ)	0.5–1.5
		Item response category functioning	An ordered threshold with the desired monotonic progression from one-step calibration to the next.
		Person separation and reliability	Person reliability>0.8; person separation index>2.0
		Item separation and reliability	Item reliability>0.8; Item separation index>3.0
		Wright map	The range of items covers the range of person distribution, and the mean for person and item should be approximately around the same location.
		Person-item map	The range of item distribution covers the range of person distribution.
		Test information function	> 70% of that of the original scale.
Validity-internal structure	CTT	Factor loading	>0.4
		Item-total correlation	*p* > 0.05
		Cronbach’s α coefficient after removing an item	The coefficient does not increase significantly.
		Items correlation with age, gender, and continent	<0.1
		Items correlation with AWS	0.3–0.5
		Confirmatory factor analysis	χ2/df should not be statistically significant; CFI > 0.9; RMSEA<0.08
	Rasch analysis	Uni-dimensionality	The first contrast of the residual >40% of the variance.
		Local independence	The standardized residual correlations <0.2
Validity-relations to others	CTT	Correlation with the MBI-GS	>0.95
		Correlation with the AWS	0.3–0.5
Fairness/equivalence	Rasch analysis	Differential item functioning	<0.5
	CTT	Multi-group confirmatory factor analysis	ΔCFI ≤0.02 and ΔRMSEA ≤0.03 for the model of measurement weights; ΔCFI ≤0.01 and ΔRMSEA ≤0.015 for the model of measurement intercepts.

CTT, classic test theory; AWS, area of worklife scale.

#### 2.3.1 Reliability/precision evidence

Reliability refers to the consistency of scores across instances of the testing procedure. In CTT, we calculated Cronbach’s alpha coefficients and omega coefficients for internal consistency. The recommended criteria for Cronbach’s α and omega coefficients are above 0.7, with values above 0.8 being more satisfactory (Nunnally and Bernstein, 1994; de Vet et al., 2017). In addition, we examined floor effects (i.e., the percentage of respondents who reported the lowest possible score) and ceiling effects (i.e., the percentage of respondents who reported the highest possible score). Floor and ceiling effects are considered problematic if more than 15–20% of respondents report either the best or worst possible score (Terwee et al., 2007). This indicates that the measure is unable to discriminate between respondents at either extreme of the scale, suggesting a limited instrument range and measurement inaccuracy.

In Rasch analysis, we tested the following: (1) item fit of information-weighted fit statistic (infit) mean square (MNSQ) and outlier-sensitive fit statistic (outfit) MnSq; (2) item response category functioning; (3) person reliability and separation;(4) item reliability and separation; (5) wright map; (6) person-item map; and (7) test information function. Item fit is defined by the consistency of responses to the item with the overall pattern of responses to the other items by the sample of respondents (Tennant and Conaghan, 2007). MNSQ is defined such that the model-specified uniform value of randomness is 1.0 (Tennant and Conaghan, 2007). An item was considered a misfit if both infit MNSQ and outfit MNSQ were > 1.5. Item response category functioning with the 7-scale response was evaluated using logit (an interval scale). An ordered threshold with the desired monotonic progression from one-step calibration to the next was considered acceptable (Tennant and Conaghan, 2007). Person and item reliability represent the measurement precision of an instrument. Person reliability (ranging from 0 to 1) above 0.8 and a person separation index above 2.0 imply that the instrument is sensitive enough to distinguish between high and low performers (Blazeby et al., 2002). Similarly, an item reliability above 0.8 (ranging from 0 to 1) and an item separation index above 3 represent an acceptable level of separation between the more endorsable and less endorsable items (Altman and Bland, 1997). Wright map of the whole scale shows whether the range of items covered the range of person distribution, and the mean for person and item should be approximately around the same location (Lozano Rojas et al., 2009). If so, this map visually provides evidence that the scale includes a sufficient number of items to measure persons at all possible lowest and highest score and can precisely differentiate between persons who were near the mean score. Similar to wright map, the person-item map in each subscale also visually provided evidence to see whether the range of item distribution covered the range of person distribution. The test information function can be viewed as a mathematical statement of the precision of measurement at each level of the given construct (Masters, 1982; American Educational Research Association, 2014). The reduction of items usually leads to information loss. We calculated the information value based on data within the range of the latent trait level (θ) of (−3, 3) (Baker, 2001). It is recommended that information of a shortened scale should reach at least 70% of that of the original scale (Baker, 2001).

#### 2.3.2 Validity evidence

Validity refers to the extent to which a measure achieves the purpose for which it is intended [29]. Under the Standards, validation involves accumulating evidence from five sources: (1) content; (2) response processes; (3) internal structure; (4) relations to other variables; and (5) consequences of testing (Streiner et al., 2015). In the development of the 16-item MBI-GS, the content validity and response processes (such as time) have been validated (Maslach et al., 1996). Consequences of testing arise from the test’s sensitivity to characteristics or from the test’s failure to fully represent the intended construct (Streiner et al., 2015). This is usually used in education examinations. Therefore, our validity testing mainly focused on internal structure validity and relation to other variables (convergent validity) in this study.

With regard to the internal structure, we collected evidence from CTT: (1) factor loadings within each subscale from exploration factor analysis with promax rotation for large sample (>0.4) (Nunnally and Bernstein, 1994), (2) the corrected item-total correlation is statistically significant (*p* < 0.05), (3) Cronbach’s α coefficient of the scale increased if a particular item is deleted (Nguyen et al., 2014; Huang et al., 2017). In Rasch’s analysis, we collected evidence: (1) the uni-dimensionality: the first contrast of the residual should explain more than 40% of the variance (Leung et al., 2014); (2) local independence that the standardized residual correlations among the items were recommended to be <0.2 (Tennant and Conaghan, 2007). Finally, we conducted confirmatory factor analysis (CFA) of the one-factor structure and the three-factor structure. The primary parameter usually used to appraise the model fit of CFA is χ^2^/df (< 2), but χ^2^/df is sensitive to sample size with a larger sample size decreasing the *p*-value where there may only be a trivial misfit (Babyak and Green, 2010). Other model fit indicators thus need to be taken into account to assess model adequacy, such as comparative fit index (CFI) and root mean square error of approximation (RMSEA) in this study (Kline, 2011). CFI values >0.90 and RMSEA values <0.08 suggest a good fit. We used a bias-corrected bootstrapping approach based on 2000 re-samples in analysis (Kline, 2011).

With regard to relations to other variables, we tested the MBI-GS9 with both criteria and conceptually related constructs. The correlation between the scale score of the shortened version and the full-length version was recommended to be above 0.95 (Harel et al., 2019). We analyzed the bivariate associations (Pearson’s correlation coefficient) between the MBI-GS9, the MBI-GS, and the six subscales of the AWS. The correlation between the MBI-GS9 and MBI-Gs was corrected with Levy’s adjustment (Levy, 1967). The correlations with instruments measuring related but dissimilar constructs (the AWS in this study) should be lower approximately 0.30–0.50 (Prinsen et al., 2018).

#### 2.3.3 Fairness/equivalence evidence

Fairness refers to responsiveness to individual characteristics and testing contexts so that test score yields valid interpretations for intended users (American Educational Research Association, 2014). The equivalence of the construct being assessed is a central issue in fairness. We analyzed equivalence at both item level and scale level. At the item level, we analyzed differential item functioning (DIF) to assess whether items are invariant across gender, age, and continent groups, with the value of 0.5 or higher indicating substantial DIF (i.e., invariance) across groups (Leung et al., 2014).

At scale level, we conducted multi-group comparisons across subgroups of gender, age, and continent, respectively. Model 1 was the primary step to see whether the instrument had the same latent structure in different groups. We then tested Model 2 and Model 3 to see whether meant factor loading and latent means were equivalent across different groups (Vandenberg and Lance, 2000). We used the following criteria to determine measurement invariance: ΔCFI ≤0.02 and ΔRMSEA ≤0.03 for the test of Model 2 (measurement weights) against Model 1 (unconstrained) and ΔCFI ≤0.01 and ΔRMSEA ≤0.015 for the test of Model 3 (measurement intercepts) against Model 2 (Rutkowski and Svetina, 2014).

## 3 Results

Following statistical analysis plan listed above, we tested the psychometric properties of the MBI-GS9 and the MBI-GS in this section, which answered the aforementioned research question and supported two hypotheses. Overall, the MBI-GS9 showed satisfactory reliability and validity and was comparable with the MBI-GS.

### 3.1 Results of reliability/precision evidence

#### 3.1.1 Cronbach’s α and omega coefficients

The Cronbach’s α and omega coefficients of three subscales in both the MBI-GS and the MBI-GS9 were satisfactory (Table 3), with values ranging from 0.84 to 0.91 against the recommended value of 0.7. The Cronbach’s α and omega coefficients did not show a significant difference. The Cronbach’s α coefficient in exhaustion slightly decreased in MBI-GS9 from 0.88 to 0.84 but slightly increased in efficacy from 0.89 to 0.91, with all coefficients approximately 0.9. Therefore, the internal consistency of the MBI-GS9 was satisfactory and comparable with the MBI-GS.

**Table 3:** Psychometric properties of the MBI-GS and the MBI-GS9 at scale level.

		CTT		Rasch				
	Subscale	Cronbach’s α coefficient	Omega coefficient	Person reliability	Person separation	Item reliability	Item separation	Uni-dimensionality
The MBI-GS	Ex	0.88	0.84	0.84	2.28	0.99	12.95	65.8%
	Cy	0.89	0.89	0.76	1.76	1.00	28.45	66.6%
	Ef	0.89	0.91	0.83	2.20	1.00	34.31	63.1%
The MBI-GS9	Ex	0.84	0.88	0.80	1.99	0.99	9.71	71.2%
	Cy	0.89	0.89	0.84	2.26	1.00	45.49	76.7%
	Ef	0.91	0.90	0.90	3.04	1.00	74.53	82.0%

Exhaustion, Ex; Cynicism, Cy; Efficacy, Ef; CTT, classic test theory.

#### 3.1.2 Floor/ceiling effect

Regarding the floor/ceiling effect, for three dimensions of the MBI-GS9, 6, 17.2, and 0.8% achieved the lowest possible score (0) and 1, 0.9, and 14.6% the highest possible score (6) on three subscales, respectively. For the three dimensions of MBI-GS, 3.1, 11.6, and 0.5% achieved the lowest possible score (0) and 0.8, 0.5, and 7.6% the highest possible score (6) on three subscales in the MBI-GS, respectively. Therefore, the MBI-GS9 did not the show floor/ceiling effect as did MBI-GS.

#### 3.1.3 Item fit

**Figure 1:**
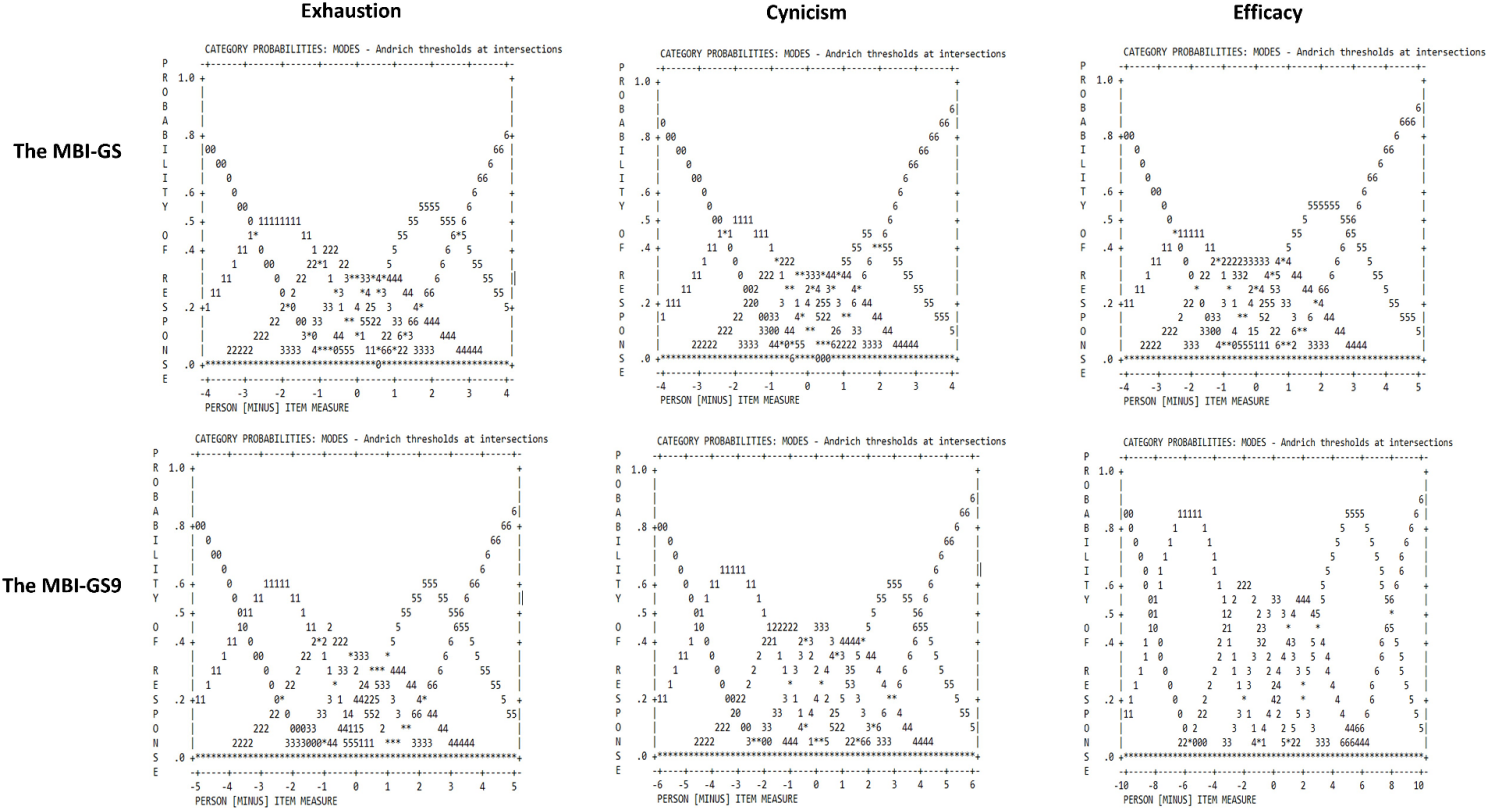
Response category functioning of the MBI-GS and the MBI-GS9.

In Rasch analysis, psychometric properties of each of the 16 items (Table 4) indicated two misfit items with both infit and outfit MNSQ>1.5, which were Cy4 (I have become more cynical about whether my work contributes anything.) and Ef1 (I am able to effectively solve problems that arise in my work). Infit MNSQ above 1.5 meant the item was more difficult, and Outfit MNSQ above 1.5 meant the variance was more than the model expected. All nine items in MBI-GS9 showed good item fits. In Figure 1, the response category functioning with the 7-scale response showed an ordered threshold with the desired monotonic progression from one-step calibration to the next. The curves showed relatively discrete peaks for each response option in the MBI-GS9 than in the MBI-GS. However, the interval between step calibrations for the #3, #4, and #5 response options in both scales was much smaller than the recommended Rasch-Andrich thresholds of a 1.4 to 5 logit interval (Linacre, 1999). Overall, the MBI-GS9 had satisfactory psychometric properties at the item level was and better than the MBI-GS in this regard.

**Table 4:** Psychometric properties of 16 items (item level).

	CTT							Rasch		
Item No.	Factor loading	Corrected item-total correlation	Cronbach’s α after removing the item	Correlation with gender	Correlation with age	Correlation with continent	Correlation with AWS	Infit MNSQ	Outfit MNSQ	Category function with disordered threshold
Ex1	0.78	0.84	0.85	0.03	−0.01	−0.21	From −0.28 to −0.57	0.87	0.86	No
Ex2	0.77	0.81	0.86	0.07	−0.04	−0.17	From −0.24 to −0.58	1.09	1.06	No
**Ex3**	**0.79**	**0.86**	**0.85**	**0.04**	**−0.09**	**−0.18**	From **−0.30 to − 0.49**	**0.83**	**0.81**	No
**Ex4**	**0.75**	**0.78**	**0.87**	**0.03**	**−0.02**	**−0.12**	From **−0.31 to − 0.43**	**1.25**	**1.25**	No
**Ex5**	**0.76**	**0.84**	**0.85**	**0.01**	**−0.05**	**−0.05**	From **−0.32 to − 0.55**	**0.92**	**0.91**	No
Cy1	0.80	0.83	0.86	−0.04	−0.05	−0.04	From −0.34 to −0.42	1.01	0.97	No
Cy**2**	**0.84**	**0.88**	**0.84**	**−0.03**	**−0.04**	**−0.00**	From **−0.39 to − 0.42**	**0.68**	**0.69**	No
Cy**3**	**0.80**	**0.86**	**0.86**	**−0.03**	−**0.05**	**0.01**	From **−0.21 to − 0.30**	**0.85**	**0.87**	No
Cy4	0.56	0.72	0.90	−0.08	−0.08	−0.01	From −0.33 to −0.45	2.08	2.07	No
Cy**5**	**0.85**	**0.88**	**0.84**	**−0.03**	**−0.04**	**−0.05**	From **−0.30 to − 0.43**	**0.72**	**0.67**	No
Ef1	0.69	0.71	0.90	−0.01	0.01	−0.13	From 0.14 to 0.28	1.54	1.72	No
Ef2	0.75	0.78	0.89	−0.02	0.12	−0.14	From 0.13 to 0.38	1.30	1.31	No
Ef**3**	**0.91**	**0.88**	**0.86**	**0.01**	**0.07**	**0.31**	From **0.12 to 0.19**	**0.59**	**0.56**	No
Ef**4**	**0.87**	**0.86**	**0.87**	**0.02**	**0.05**	**−0.08**	From **0.19 to 0.58**	**0.72**	**0.77**	No
Ef**5**	**0.88**	**0.86**	**0.87**	**0.01**	**0.10**	**−0.24**	From **0.15 to 0.36**	**0.72**	**0.72**	No
Ef6	0.80	0.80	0.88	0.02	0.06	−0.19	From 0.25 to 0.32	1.08	1.10	No

Exhaustion, Ex; Cynicism, Cy; Efficacy, Ef; CTT, classic test theory. Correlations among items in MBI-GS9 were bold.

#### 3.1.4 Person and item separation and reliability

In Rasch analysis at the scale level, listed in Table 3, Rasch person reliabilities of the MBI-GS9 were all above 0.8 (with person separation index >2). The person separation index of Cynicism in the MBI-GS was 1.76, which was below 2. The Rasch item reliabilities in the MBI-GS9 and the MBI-GS were high all approximately 1.0 (with item separation index >3). These indicated that the MBI-GS9 was able to distinguish between the high and low performers, with an acceptable separation between the more endorsable and less endorsable items.

#### 3.1.5 Wright map

**Figure 2:**
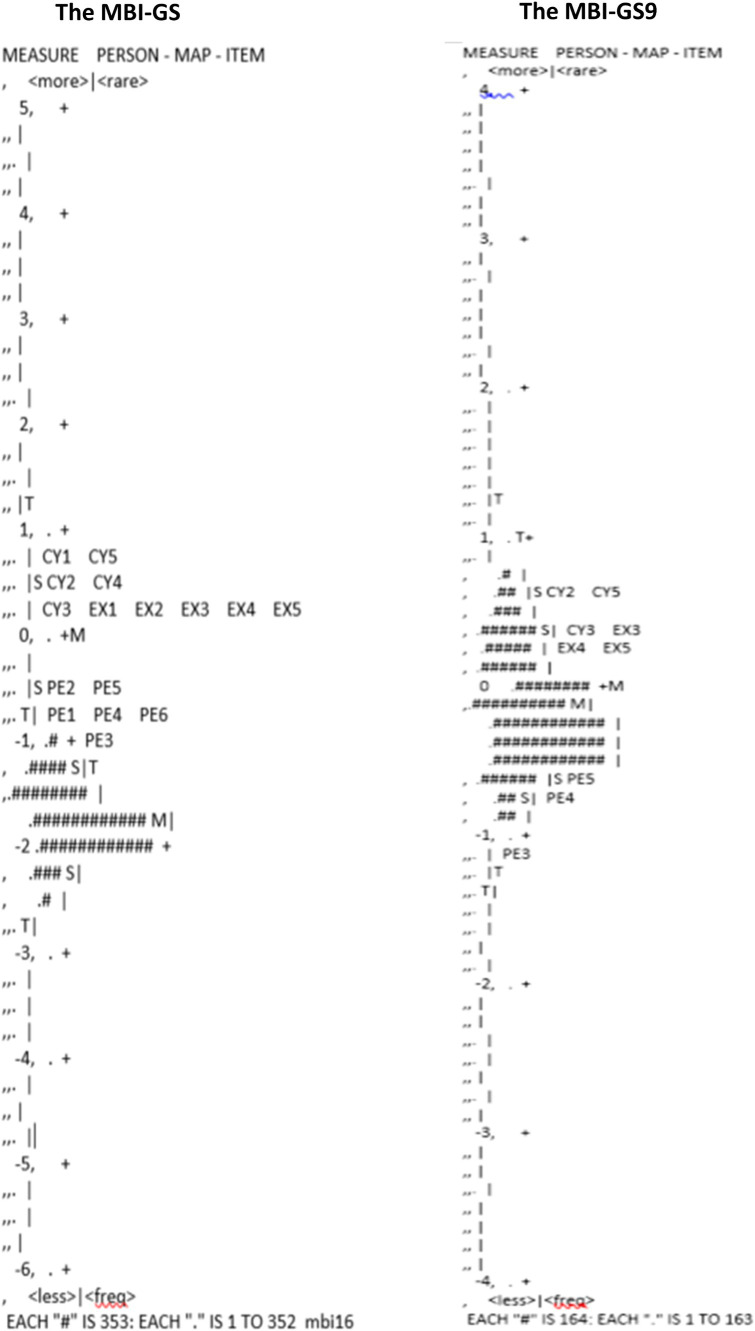
Wright maps of the MBI-GS and the MBI-GS9.

Figure 2 shows the Wright maps for the MBI-GS and the MBI-GS9. The left side of the map showed the distribution of respondents in the sample arranged along a hierarchy by person burnout. The right side of the map showed the array of items ordered by item endorsability. Persons at the top have the highest level of burnout, and items at the top of the map are the most unlikely to endorse (Lozano Rojas et al., 2009). Both the MBI-GS and the MBI-GS9 showed clear item difficulty hierarchy on the right side. For the MBI-GS9, the person mean was near the item mean, and item distribution covered the range of person distribution. Therefore, the MBI-GS9 had a sufficient number of items to measure persons at all possible lowest and highest scores and can precisely differentiate between persons who were near the mean score, better than the MBI-GS.

#### 3.1.6 Person-item map

**Figure 3:**
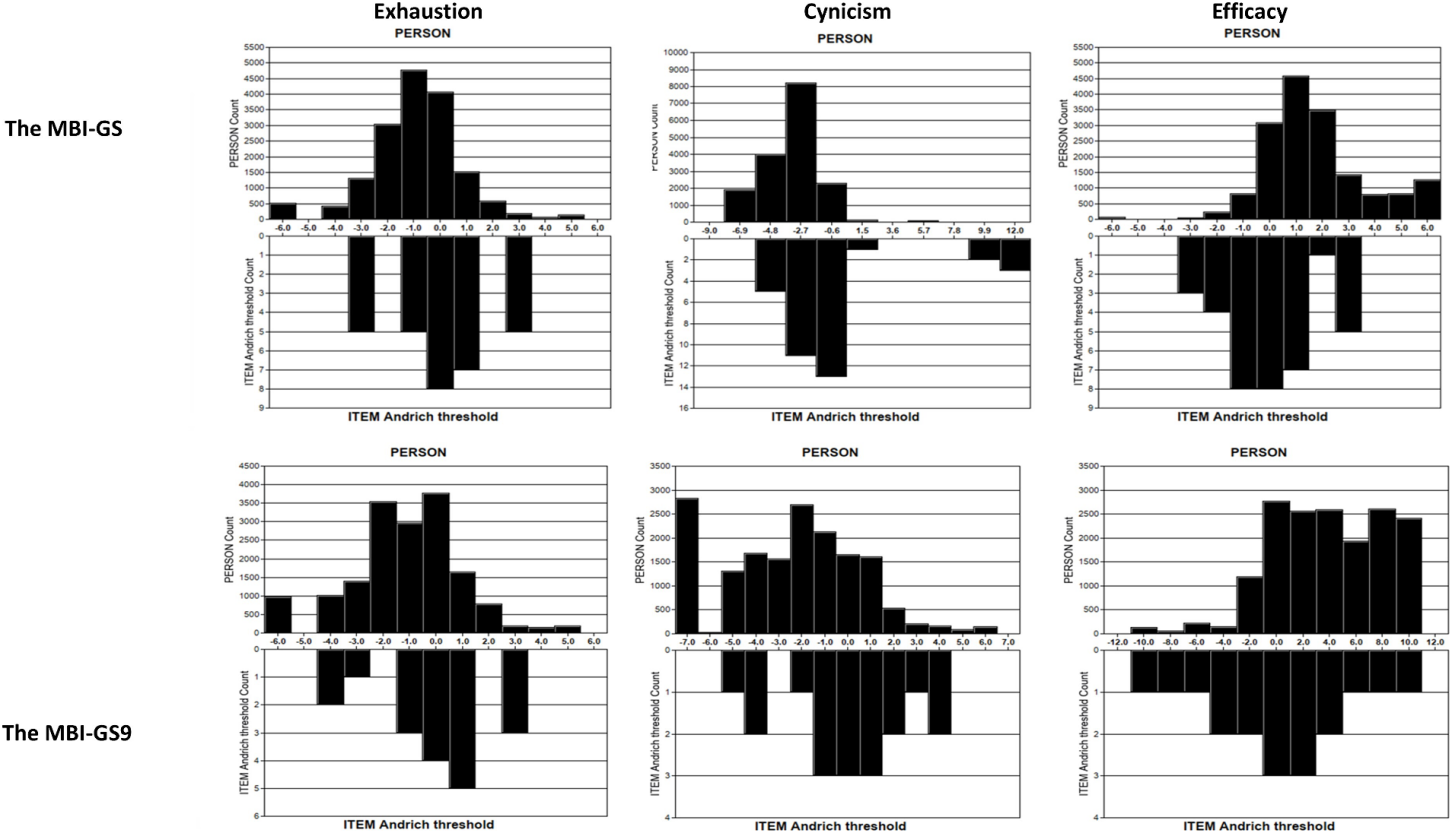
Person-item threshold distribution plot of the MBI-GS and the MBI-GS9.

Figure 3 shows the spread of person-item locations in three subscales. In the MBI-GS9, the ranges of item thresholds (the lower half) generally covered the ranges of the measured constructs (the upper half). Only a few extreme lowest values in Exhaustion and Cynicism were below the sensitivity that could be captured by the MBI-GS9. In the MBI-GS, the ranges of item thresholds covered the ranges of the measured constructs regarding exhaustion and cynicism, but for efficacy in the MBI-GS, the spread of persons with high values exceeded the right side of the item range, indicating a ceiling effect. Overall, items in the MBI-GS9 better fit the measured construct (burnout in this study) than the MBI-GS, providing a more precise assessment.

#### 3.1.7 Test information function

Supplementary Appendix 1 shows the test information of the MBI-GS and the MBI-GS9. The main bodies were generally resembled. We usually want the test information function to peak at the latent trait level (θ) of 0, where the mode of the sample is norm-referenced tests. In the MBI-GS, the peak was at approximately −1.1 and was near 0 in the MBI-GS9. The test information over the latent trait level (θ) of (−3, 3) was 503.9 and 798.41 for the MBI-GS9 and the MBI-GS, respectively. The MBI-GS9 took up 63% of the test information of the MBI-GS, which is a bit under the criteria of 70% (Harel et al., 2019). Therefore, we further estimated the sample size required to compensate for the information loss. We used the comparison of the means of two dependent groups (matched pairs) in Gpower 3.0. We set α = 0.05, 1-β = 0.95, and mean difference SD = 0.40 in this study. The sample size increased as the effect size decreased. When the sample size was roughly approximately 350, the effect size was 0.2 (usually indicating limited practical applications), and when the sample size was 1,300, the effect size was 0.1 usually indicating no effect (Aspinwall and Tedeschi, 2010). Supplementary Appendix 2 shows the line figure of a sample size of effect size and mean difference under the calculation of means of two dependent groups (matched pairs).

### 3.2 Results of validity evidence

#### 3.2.1 Structure validity

With regard to the internal structure, in CTT, all items showed satisfactory properties in factor loadings (without cross-loading), item-total correlation, and Cronbach’s α coefficient after removing an item (Table 4). In Rasch’s analysis regarding the uni-dimensionality of each subscale (Table 3), the variance explained by the measure was high and above 40%. This provided a prerequisite for conducting factor analysis. The standardized residual correlations among the items in the MBI-GS9 generally met the criteria of 0.2, lower than that of the MBI-GS (Table 5). This indicated that MBI-GS9 showed better local independence. Then, we conducted CFA (Table 6) and found that the one-factor structure was fairly poorly fitted. The correlated three-factor structure had significant χ^2^/df statistics but had good RMSEA (0.07) and CFI (0.97) indexes supporting the model adequacy. The significant χ^2^/df statistic was probably caused by the large sample in this study. To confirm this, we randomly selected 200 cases out and ran the model again. The χ^2^/df statistic changed to be not significant. Therefore, a theoretically driven three-factor structure was supported.

**Table 5:** Standardized residual correlations among 16 items within three subscales.

	Ex1	Ex2	Ex3	Ex4	Ex5	
Ex1	–					
Ex2	−0.08	–				
Ex3	−0.26	−0.16	–			
Ex4	−0.34	−0.51	**−0.17**	–		
Ex5	−0.26	−0.28	**−0.30**	**−0.12**	–	
	Cy1	Cy2	Cy3	Cy4	Cy5	
Cy1	–					
Cy2	−0.01	–				
Cy3	−0.33	**−0.07**	–			
Cy4	−0.32	−0.44	−0.43	–		
Cy5	−0.22	**−0.23**	**−0.14**	−0.20	–	
	Eff1	Eff2	Eff3	Eff4	Eff5	Eff6
Ef1	–					
Ef2	−0.16	–				
Ef3	−0.28	−0.30	–			
Ef4	−0.37	−0.31	**0.04**	–		
Ef5	−0.41	−0.24	**0.04**	**0.17**	–	
Ef6	−0.16	−0.27	−0.06	−0.24	−0.29	–

Exhaustion, Ex; Cynicism, Cy; Efficacy, Ef. The correlations among items in MBI-GS9 were bold.

**Table 6:** Structure of two scales and invariance across gender, age, and continent of the MBI-GS9.

Full sample	χ^2^	df	χ^2^/df	RMSEA	CFI	Pass
1-factor model of the MBI-GS	82766.82	104	795.83	0.21	0.50	No
1-factor model of the MBI-GS9	43327.12	27	1604.70	0.31	0.53	No
3-factor model of the MBI-GS	10770.97	101	106.64	0.08	0.97	Yes
3-factor model of the MBI-GS9	2036.94	24	84.87	0.07	0.97	Yes
**Gender**						
Model 1 unconstrained	2163.76	48	45.07	0.05	0.97	Yes
Model 2 measurement weights	2185.90	54	40.48	0.05	0.97	Yes
Model 3 measurement intercepts	2389.00	65	36.75	0.05	0.97	Yes
**Age**						
Model 1 unconstrained	4078.02	210	19.41	0.03	0.95	Yes
Model 2 measurement weights	4088.38	216	18.92	0.03	0.95	Yes
Model 3 measurement intercepts	4228.37	227	18.62	0.03	0.95	Yes
**Continent**						
Model 1 unconstrained	11305.09	156	72.46	0.06	0.88	Yes
Model 2 measurement weights	11521.91	162	71.12	0.06	0.87	Yes
Model 3 measurement intercepts	13104.11	173	75.74	0.06	0.86	No

Model 1 unconstrained = configural invariance. Model 2 measurement weights = metric invariance. Model 3 measurement intercepts = scalar invariance.

#### 3.2.2 Convergent validity

With regard to relations to others, we found a very strong correlation between any of the MBI-GS9 subscales and their corresponding subscales in the MBI-GS with correlation coefficients without Levy’s adjustment ranging from 0.94 to 0.96 (Table 7). These coefficients nearly met the criteria of above 0.95. These indicated a rather gold-standard criteria validity of the MBI-GS9 against the MBI-GS. The correlation coefficients with Levy’s adjustment ranged from 0.78 to 0.84, suggesting some degree of information loss. This finding aligned with the test information results reported earlier.

**Table 7:** Relationship of the MBI-GS9, the MBI-GS, and the AWS.

	The MBI-GS9			The MBI-GS		
	Ex	Cy	Ef	Ex	Cy	Ef
Correlation with the MBI-GS						
Ex	0.95^a^ (0.78)^b^ **			-		
Cy		0.96 ^a^ (0.85)^b^ **			-	
Ef			0.94^a^ (0.84)^b^ **			-
Correlation with the AWS						
Workload	−0.54**	−0.36**	0.18**	−0.59**	−0.38**	0.22**
Control	−0.34**	−0.38**	0.29**	−0.33**	−0.40**	0.36**
Reward	−0.40**	−0.45**	0.33**	−0.40**	−0.48**	0.37**
Community	−0.38**	−0.44**	0.35**	−0.37**	−0.46**	0.41**
Fairness	−0.41**	−0.47**	0.27**	−0.41**	−0.49**	0.33**
Values	−0.34**	−0.46**	0.37**	−0.33**	−0.48**	0.43**

*P < 0.05, **p < 0.01. Exhaustion, Ex; Cynicism, Cy; Efficacy, Ef. ªCorrelation without Levy’s adjustment. ^b^Correlation with Levy’s adjustment.

The three subscales and each item were significantly correlated with the subscales in the AWS, with coefficients approximately 0.12–0.58 that generally meet the criteria of approximately 0.30–0.50 (Tables 4, 7). The correlation and regression coefficients of the MBI-GS9 were similar to that of the MBI-GS. Therefore, the MBI-GS9 had a reasonable relationship with conceptually related areas of worklife.

### 3.3 Equivalence across gender, age, and continent

#### 3.3.1 Differential item functioning

The Rasch analysis provided the DIFs of each item (Table 8). We found that the DIFs were all below 0.5 logits across almost all subgroups of gender and age. This represented a rather good generalizability in different ages and genders of the items. In examining DIF among the continent subgroups, there were few DIFs above the criterion of ≥0.5 logit. These are in accordance with the correlation analysis of each item listed in Table 4. It was found that each item had a weak correlation (less than 0.1) with gender and age but exhibited slightly stronger correlations with continent (ranging from 0.00 to 0.31).

**Table 8:** Differential item functioning across gender, age, and continent.

Item	Gender	Age	Continent
Ex1	None	None	None
Ex2	None	None	None
**Ex3**	**None**	**None**	**1–4, 0.78; 1–3, 0.79**
**Ex4**	**None**	**None**	**1–3, 0.62; 1–4, 0.74**
**Ex5**	**None**	**None**	**None**
Cy1	None	None	None
**Cy2**	**None**	**None**	**None**
**Cy3**	**None**	**None**	**None**
Cy4	None	1–5, −0.65	1–3,0.84; 1–4,0.91
**Cy5**	**None**	**None**	**1–4,1.16; 2–4,1.30; 3–4,0.83**
Ef1	None	None	1–3, −0.57; 3–3, −0.68
Ef2	None	None	None
**Ef3**	**None**	**None**	**1–2, −1.01; 1–3, −1.35; 1–4, −0.76**
**Ef4**	**None**	**None**	**1–2, 0.94; 1–3, 0.83; 1–4, 1.16**
**Ef5**	**None**	**None**	**3–4, −0.86**
Ef6	None	None	None

Those only with DIF ≥ 0.5 were listed. Age code: 1 = 18–29, 2 = 30–39, 3 = 40–49, 4 = 50–59, 5 = 60+. Continent code: 1 = North America, 2 = Europe, 3 = Others, 4 = Asia. The items in MBI-GS9 were bold.

#### 3.3.2 Multi-group confirmatory factor analysis

Furthermore, we conducted multi-group CFA to test the measurement invariance across gender, age, and continent (Table 6). The results supported that the MBI-GS9 had measurement equivalence across gender and age, supporting hypothesis 1. Only measurement intercepts of the MBI-GS9 across continents were variants, indicating people from different continents had different latent values (i.e., exhaustion, cynicism, and efficacy in this study). Thus, the hypothesis 2a was supported, and hypothesis 2b was clarified. The findings in multi-group CFA were in accordance with the DIFs in Table 8. Overall, the MBI-GS9 showed equivalent structure and factor loading in different gender, age, and continent groups and showed invariant latent values across continents.

## 4 Discussion

By employing sophisticated methods of Rasch analysis and classical test theory (CTT), this study conducted a comprehensive assessment of the psychometric properties of the MBI-GS9. The evidence of reliability and validity supported that the MBI-GS9 had satisfactory measurement properties and was comparable to the MBI-GS. The MBI-GS9 demonstrated equivalent factor structure and factor loadings across different gender, age, and continent groups, as well as equivalent latent values across continents. These findings indicate that the MBI-GS9 is a reliable and valid instrument for measuring burnout across diverse populations and settings.

### 4.1 The MBI-GS9 against the MBI-GS: scale level and item level

At scale level, the changes in the MBI-GS9 properties compared to the MBI-GS were minor. In our study, with a relatively large sample, the Cronbach’s α coefficients for MBI-GS9 ranged from 0.84 to 0.91, which were higher than those reported for 347 Peruvian teachers (0.67–0.80) (Merino-Soto et al., 2023). The MBI-GS9 showed better response category functioning and person reliability but reduced test information. As to response category functioning, three subscales of MBI-GS9 showed clearer peaks in response category functioning. Although the MBI-GS also showed an ordered threshold, the interval between step calibrations for the #3 (A few times a month), #4 (Once a week), and #5 (A few times a week) response options in cynicism was slightly overlapped. Both the MBI-GS9 and the MBI-GS failed to meet the recommended Rasch-Andrich thresholds of a 1.4–5 logit interval (Linacre, 1999). These findings suggested that the respondents were not clearly distinguishing between those response options (3–5). The researchers can collapse these overlapped response options in their use of MBI-GS9. Some studies decreased the number of scaling, which yielded a more meaningful category probability curve and in turn represented a distinct portion of the underlying construct (Linacre, 1999), and also reduced task-taking time. Nonetheless, a more detailed exploration of clinically meaningful category intervals is needed in future research in assessing burnout.

The person reliability of cynicism was 0.84 for MBI-GS9 and 0.76 for MBI-GS. The recommended ideal person reliability is 0.8 (Blazeby et al., 2002), while a person reliability lower than 0.8 indicates a need for introducing new items that can separate high and low performers or introduce a broader sample of people with extremely high and low abilities (Tennant and Conaghan, 2007). The three-item subscale of cynicism in MBI-GS9 with a person reliability of 0.84 had sufficient and appropriate items that targeted the high and low range of burnout, performing better than the full five-item cynicism subscale. This finding was also supported by Wright maps and person-item threshold distribution plots of the scale. The MBI-GS9 showed equivalent item mean and person mean, and the item distribution covered the range of person distribution.

We found that the testing information of the MBI-GS9 accounts for 63% of the testing information of the MBI-GS, close to the criteria of 70% (Harel et al., 2019). This loss of test information is expected due to item reduction (Baker, 2001). Consequently, using the 9-item version may require a larger sample size to compensate for the reduced test information. In practice, the MBI-GS9 can be used for a pilot study and an initial screening (Merino-Soto et al., 2023). To provide a suggestion for choosing which versions for users, we conducted an estimation by rigid power criteria and a mean difference SD of 0.4 derived from this large sample. Nonetheless, future studies should employ a more precise estimate based on the characteristics of the target population and the desired study power before determining which version to use.

At the item level, the study provided rich informative evidence. All original 16 items showed good psychometric properties in CTT, but in Rasch analysis, infit MNSQ and outfit MNSQ of Cy4 (I have become more cynical about whether my work contributes anything) and Ef1 (I am able to effectively solve problems that arise in my work) exceeded 1.5, which meant underfit. Underfit indicates too much variation as responses are too haphazard (i.e., hard to endorse these two items). However, all nine items in MBI-GS9 all had good item properties and lower residual correlations below 0.2. These results of item-level analysis might explain why at the scale level, the MBI-GS9 performed generally better than the MBI-GS. Overall, the item properties were satisfactory in different cultural contexts, and the two items in the MBI-GS may require further content revision in future work.

### 4.2 Measurement invariance of the MBI-GS9 across gender, age, and continent

This is the first study to test measurement invariance for the MBI-GS9 by gender, age, and social context (continent). Our results support measurement invariance in the three-factor structure with equal factor loading and equal latent means across age and gender groups. This finding was consistent with evidence from previous studies regarding measurement invariance by gender (Aboagye et al., 2018; Portoghese et al., 2018; Brady et al., 2021; Bui et al., 2022) and age (Tang, 1998; Fernández-Arata et al., 2015; Juarez Garcia et al., 2020; Brady et al., 2021; Bravo et al., 2021). This meant the understanding of the MBI-GS9 among participants of different genders and ages was consistent, and there was no systematic bias associated with measurement factors.

We also found that the MBI-GS9 had measurement invariance in a three-factor structure with equal factor loading across continents. Several studies that validated the MBI-GS (Marais et al., 2009; Fernández-Arata et al., 2015; Juarez Garcia et al., 2020; Bravo et al., 2021) all supported the theoretically based three-factor structure. These indicated that the theoretical construct of burnout is rather stable. However, measurement invariance in latent means across continents was not established. The latent means were unequal across continents, indicating that people from different continents tended to have a systematic difference in reporting the MBI-GS9. The cross-continent differences in latent factor means may be in part due to multiple issues related to translation as indicated in some existing studies (Squires et al., 2014). While confirming the findings from multi-group CFA, the DIFs further revealed items that had statistically significant cross-continent differences, including EX3 (i.e., feel tired in the morning…), Ex4, Cy5, Ef3, Ef4, and EF5. A study using the MBI-educational survey indicated item-level differences between U.S. and Jamaica samples for items Ex2 (I feel used up at the end of the work day) and Ef9 (I feel I am positively influencing other people’s lives through my work) (Denton et al., 2013). Another study using the MBI-GS indicated item-level differences between Dutch and Greek samples for items Ex1 and Ex2, and Cy5 as well as Ex6 (Xanthoupoulou et al., 2013). Peruvian sample showed that Cy3 (just want to do my job and not be bothered) of the MBI-GS9 was found to be ambiguous (Schutte et al., 2000). Combined with the listed studies and our findings, we found different items had variances in different cultural samples. Thus, it is not advisable to assume that the items remain invariant without checking when conducting cross-cultural comparisons (Denton et al., 2013; Squires et al., 2014).

### 4.3 Implication for burnout practice and limitation

One of the major strengths of this study was a large from various professional settings and diverse sample with different genders, ages, and continents, which ensured the generalizability of our findings. Another strength was the use of Rasch analysis combined with CCT methods, which allowed a critical comprehensive psychometric analysis. As MBI-GS9 is occupational neutral, the findings and strengths of this study support the use of MBI-GS9 across various age and gender groups and various professional settings. However, the inequivalent latent values suggest the need for further cross-cultural exploration. Investigators may need to employ more rigorous cross-cultural adaptation methods when attempting to conduct comparisons (Squires et al., 2014).

However, several limitations have to be noted. First, due to missing information on occupation, we did not compare the measurement invariance across occupations. Burnout is an important occupational health indicator. The MBI-GS9 has been used in many different occupations and has shown good validity. We would recommend to test the measurement equivalence of the MBI-GS9 across various occupations to obtain direct evidence. Second, we did not explore the cutoff even though the practitioners might have great interest, and some studies did conduct this analysis. The MBI developers have always protested that a cutoff value makes burnout more such as a medical disease, rather than a continuum of experience. Since the MBI-GS is a self-report measure, emphasis on cutoff may potentially increase social desirability response bias. It is recommended to describe the level of burnout within the context of the study population to define low, moderate, and high levels (Maslach and Leiter, 2016). If future burnout is defined as a certain kind of disorder, further studies aimed at resolving this issue would be desirable afterward.

## 5 Conclusion

In conclusion, this study utilized Rasch analysis and classical test theory (CTT) under the framework of the Standards to evaluate the psychometric properties and measurement invariance across age, gender, and continents of the MBI-GS9. The findings indicate that the MBI-GS9 possesses satisfactory measurement properties and is comparable to the MBI-GS in terms of reliability and validity while retaining a three-factor structure. The MBI-GS9 demonstrated equivalent structure and factor loadings across different gender, age, and continent groups, as well as invariant latent values across continents. However, it is important to note that a large sample size may be necessary to compensate for the information loss in the MBI-GS9. Overall, the MBI-GS9 emerges as a reliable and valid alternative to the MBI-GS, particularly when used in large samples across different age and gender groups. For cross-cultural comparisons, it is crucial to initially examine the equivalence of different language versions at both the item level and scale level. This ensures the validity and comparability of results across diverse cultural contexts.

## Data availability statement

The datasets presented in this article are not readily available because the data is kept in the Department of Psychology, Acadia University, and the dean should be contacted for approval. Requests to access the datasets should be directed to ML, michael.leiter@acadiau.ca.

## Ethics statement

The studies involving humans were approved by the Department of Psychology, Acadia University. The studies were conducted in accordance with the local legislation and institutional requirements. Written informed consent for participation in this study was provided by the participants’ legal guardians/next of kin.

## Author contributions

AW: Writing – review & editing, Writing – original draft, Visualization, Validation, Methodology, Formal analysis, Data curation. YD: Writing – review & editing, Data curation, Conceptualization. PN: Writing – review & editing, Supervision, Data curation, Conceptualization. ML: Writing – review & editing, Supervision, Resources, Methodology, Data curation. CE: Writing – review & editing, Validation, Supervision, Project administration, Methodology, Data curation, Conceptualization.

## Footnotes

^1^ https://www.winsteps.com/index.htm
