# Supplementary figures and images for: Validation of the Maslach Burnout Inventory-General Survey 9-item short version: psychometric properties and measurement invariance across age, gender, and continent
Source: Front Psychol. 2024 Jul 16;15:1439470. doi: 10.3389/fpsyg.2024.1439470 (PMC11286593; doi:10.3389/fpsyg.2024.1439470)

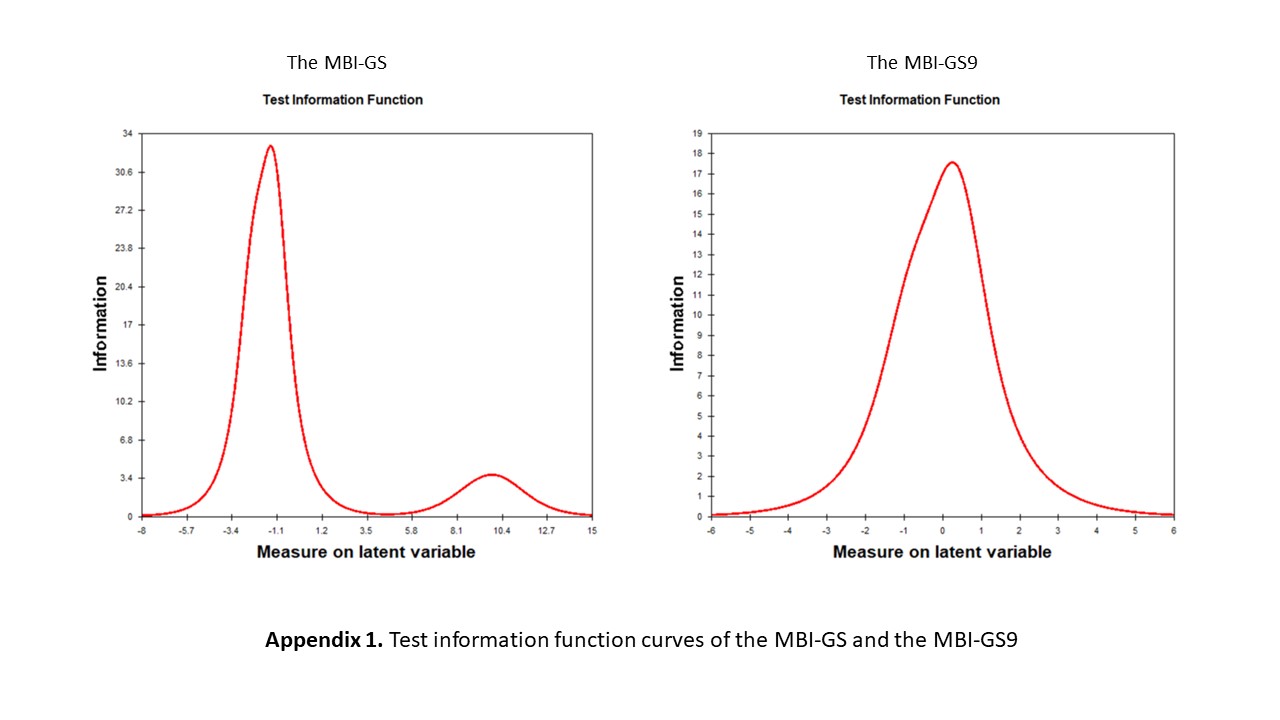

Supplement: Supplementary file 1 [file Image_1.jpg]

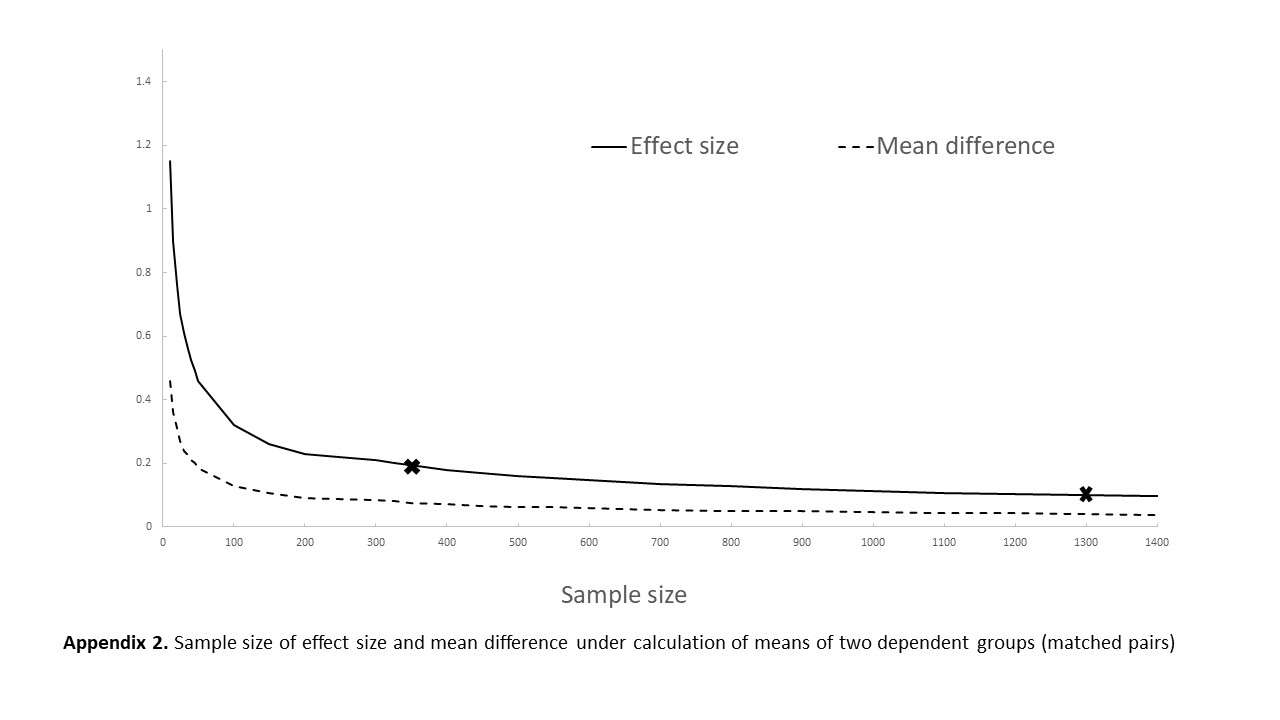

Supplement: Supplementary file 2 [file Image_2.jpg]
